# Transforming Growth Factor β/activin signalling induces epithelial cell flattening during *Drosophila* oogenesis

**DOI:** 10.1242/bio.201410785

**Published:** 2015-02-13

**Authors:** Isabelle Brigaud, Jean-Luc Duteyrat, Julien Chlasta, Sandrine Le Bail, Jean-Louis Couderc, Muriel Grammont

**Affiliations:** 1 Université Lyon 1, Lyon and Centre de Génétique Moléculaire et Cellulaire, CNRS UMR 5534, Villeurbanne, France; 2 Laboratoire Joliot Curie, CNRS, ENS Lyon, Université Claude Bernard Lyon 1, Université de Lyon, Lyon, France; 3 CNRS 6293, Clermont University, Inserm U1103, UMR GReD, UFR Médecine, Clermont-Ferrand F-63001, France

**Keywords:** Epithelial cell flattening, Adherens junction, TGFβ

## Abstract

Although the regulation of epithelial morphogenesis is essential for the formation of tissues and organs in multicellular organisms, little is known about how signalling pathways control cell shape changes in space and time. In the *Drosophila* ovarian epithelium, the transition from a cuboidal to a squamous shape is accompanied by a wave of cell flattening and by the ordered remodelling of E-cadherin-based adherens junctions. We show that activation of the TGFβ pathway is crucial to determine the timing, the degree and the dynamic of cell flattening. Within these cells, TGFβ signalling controls cell-autonomously the formation of Actin filament and the localisation of activated Myosin II, indicating that internal forces are generated and used to remodel AJ and to promote cytoskeleton rearrangement. Our results also reveal that TGFβ signalling controls Notch activity and that its functions are partly executed through Notch. Thus, we demonstrate that the cells that undergo the cuboidal-to-squamous transition produce active cell-shaping mechanisms, rather than passively flattening in response to a global force generated by the growth of the underlying cells. Thus, our work on TGFβ signalling provides new insights into the mechanisms through which signal transduction cascades orchestrate cell shape changes to generate proper organ structure.

## INTRODUCTION

Epithelial morphogenesis, which includes any process requiring a deformation within an epithelial cell sheet, is a key precondition for proper tissue and organ development, as well as for their repair during adult life. Epithelial cells are, typically, polarized cells with apical, lateral and basal domains. Morphogenetic processes usually affect the relative sizes of these domains and, temporally or permanently, modify the expression patterns of components of these domains (St Johnston and Sanson, 2011). In particular, the lateral domain contains the intercellular junctions, such as adherens junctions (AJ), that physically couple adjacent cells. Through its interaction with the actomyosin network and in response to its contractibility, AJ are often remodelled during morphogenesis (Bertet et al., 2004; Blankenship et al., 2006; Harris and Peifer, 2007). The mechanisms that regulate AJ remodelling remain unclear, especially with respect to the role of signalling pathways in their anchoring, turnover or link with the cytoskeleton. One of the model systems to study the interactions between signalling pathways and AJ remodelling is the follicular epithelium of the *Drosophila* ovary (Deng and Bownes, 1997; Peri et al., 2002; Yakoby et al., 2008; Zartman et al., 2009).

**Fig. 1.**
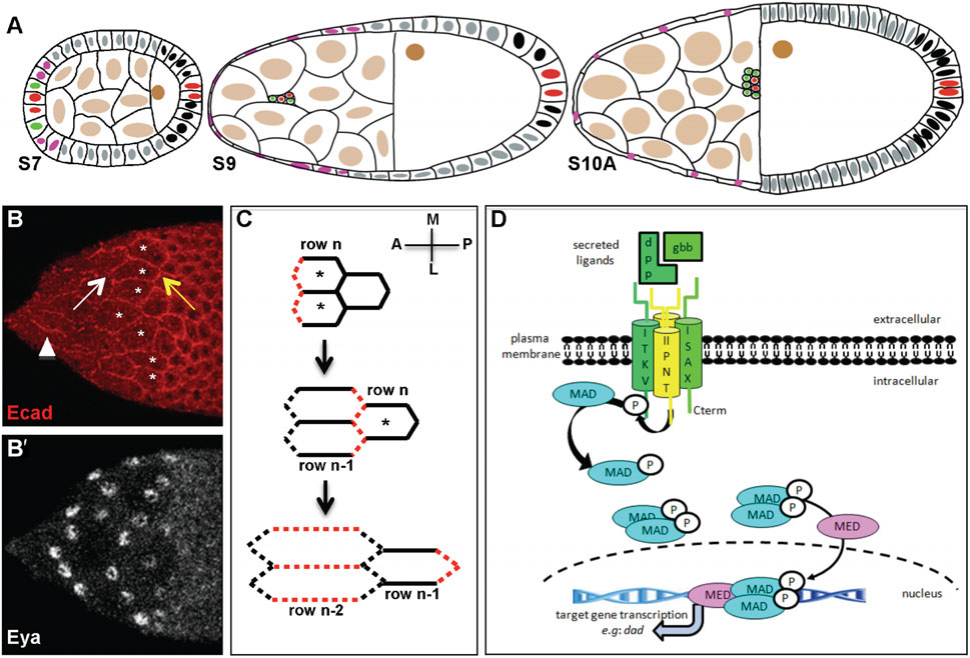
Cell flattening during *Drosophila* oogenesis. In all figures, anterior is to the left. (A) Schematic of a stage 7 (S7), 9 (S9) and 10A (S10A) follicles. The arrangement and identities of the oocyte (nucleus coloured in brown), nurse cells (light brown), main body follicular cells (grey), polar cells (red), border cells (green), stretched cells (pink) and posterior cells (black) are shown. (B–B′) A mid stage 9 follicle showing adherens junction (AJ) remodelling of the StC. The asterisks indicate flattening StC. Ecad is no longer detected at the three-cell junctions between flattened and flattening StC (white arrow), but is visible at the three-cell junctions between flattening and cuboidal cells (yellow arrow). Ecad is temporally maintained at the A/P oriented junctions (arrowhead). Eya expression is detected in the nuclei of the flattened and flattening StC. (C) Schematic of AJ disassembly in StC during stage 9. AJ of two StC rows are represented at three stages of flattening: row n: flattening StC (asterisks); row n-1: flattened StC; row n-2: fully flattened StC. The AJ that are disassembling are in red. (D) Schematic representation of the TGFβ/BMP signalling pathway in Drosophila. Activation starts with the binding of a homo or heterodimer ligand to a tetrameric complex of two types II and two types I receptors. Under binding, the type II receptor phosphorylates the type I receptor, which in turn activates Mad. Two phosphorylated Mad associate with Med and translocate to the nucleus (Wharton and Derynck, 2009).

The *Drosophila* follicle consists of an epithelial monolayer of cuboidal somatic follicular cells surrounding an oocyte and 15 nurse cells. The follicular cells progressively differentiate into various sub-populations that experience the different cell-shape changes and/or migrations required to pattern the future eggshell (Fig. 1A) (Horne-Badovinac and Bilder, 2005). The cell shape changes start at stage 9 with about 50 stretched cells (StC) that flatten dramatically over the nurse cell compartment, and with the border cells that delaminate from the epithelium and migrate posteriorly between nurse cells. Concomitantly, the posterior cells and the main body follicular cells become columnar around the growing oocyte (Kolahi et al., 2009). At the beginning of stage 10A, the boundary between the flattened StC and the columnar cells is aligned with the nurse cell-oocyte interface.

The flattening process of the StC leads to anisotropic shape, with cells elongating more along the anterior-posterior (A/P) axis than the medio-lateral (M/L) axis (Grammont, 2007; Kolahi et al., 2009). It begins at the anterior pole and spreads row by row in an anterior to posterior wave, such that all the cells located at the same position relative to the A/P axis are at a similar phase of cuboidal-to-squamous transition (Fig. 1B,C). Stretched cell shape changes require extensive adherens junctions (AJ) remodelling with the spatial and temporal regulation of two apicolateral AJ components, DE-cadherin (Ecad, encoded by *shotgun*) and β-catenin (encoded by *armadillo*), and with changes in Actin cytoskeleton dynamics through the activity of the non-muscular Myosin II (encoded by the *zipper* (*zip*) and *spaghetti squash* (*sqh*) genes). The mechanisms that drive AJ remodelling and cytoskeleton rearrangement are still unclear. From morphometric analyses and manipulation of the growth of the underlying germline, it has been proposed that StC flatten in response to the tension generated by this growth through a “compliant” flattening process (Kolahi et al., 2009). This is counterbalanced by the analyses from the Notch pathway as well as the *hindsight* and *tao* genes, which suggest the presence of active and cell-autonomous mechanisms in the StC to allow their flattening to occur (Gomez et al., 2012; Grammont, 2007). First, the Fringe-dependent Notch pathway controls the expression patterns of Ecad, Arm, Zip and Sqh (Grammont, 2007). Second, the *hindsight* gene, a transcription factor that putatively negatively regulates JNK signalling, regulates Ecad and Arm expression level (Melani et al., 2008). Third, the *tao* gene, which is known to activate the stress-responsive MAPK pathway in mammalian cells and the Salvador-Warts-Hippo pathway in *Drosophila*, promotes the endocytosis of the cell adhesion molecule Fasciclin 2, the *Drosophila* N-CAM (neural cell adhesion molecule), from the lateral membrane (Gomez et al., 2012).

Another cell-autonomous possible regulator of the cuboidal-to-squamous transition could be the Transforming Growth Factor/Bone Morphogenetic Protein (TGFβ/BMP) pathway. In *Drosophila*, TGFβ ligands are encoded by the *decapentaplegic* (*dpp*), *glass bottom boat (gbb*) and *screw* genes; the type I receptors by the *saxophone* (*sax*) and *thick veins* (*tkv*) genes; the type II receptors by the *punt* and *wishful thinking* (*wit*) genes; and the Smads by the *Mothers against dpp* (*Mad*) and *Medea* (*Med*) genes (Fig. 1D) (Miyazawa et al., 2002; Wharton, 1995). It has been shown that the TGFβ pathway is active in the StC during stage 9 (Guichet et al., 2001; Muzzopappa and Wappner, 2005; Twombly et al., 1996). Indeed, the *dpp* mRNA, the phosphorylated form of the Mad and the ß-galactosidase activities from enhancer-trap elements inserted at the *dpp* and at the *Daughter against dpp* (*Dad*) loci, are specifically detected in the StCs at stage 9. However, no study has been undertaken to determine whether the activation of this pathway in these cells during their flattening is functionally relevant.

In the article, we make a comprehensive study of the contribution of the canonical TGFβ pathway actors during StC flattening by genetically manipulating their expression levels. We find that TGFβ expression from the StC is required for the cuboidal-to-squamous follicular cell transition. We show that the activation of the TGFβ pathway within these cells autonomously regulates the dynamic and the degree of the flattening, by acting on AJ remodelling and cytoskeleton rearrangement. We show that one of the targets of the TGFβ signalling is the Notch gene and that restoring N activity can partially rescue impaired TGFβ signalling. Finally, our results indicate that the temporal regulation of TGFβ activity is crucial to set up the timing of StC flattening, demonstrating that StC actively participate to their flattening by producing internal forces.

## MATERIALS AND METHODS

### *Drosophila* stocks and crosses

The mutant stocks used are *tkv^a12^* FRT40A, *tkv^8^* FRT40A, *tkv^7^* FRT40A, *Mad^1-2^* FRT40A, *Mad^12^* FRT40A, *Mad^8-2^* FRT40A, FRT82B *punt^Δ61^*, FRT82B *punt^135^*, FRT82B *punt^10460^*, FRT82B *Med^8^*, FRT82B *Med^13^*, FRT82B *Med^4^*, FRTG13 *gbb^1^*, FRTG13 *sax^4^*, *wit^G15^* FRT2A, *Smox^MB388^* FRT19A, FRTG13 *babo^52^*, dpp^d12^ FRT40A, dpp^d14^ FRT40A, Dl^rev10^ FRT82B and TRIP.dpp^JF01371^ (Bangi and Wharton, 2006; Cordero et al., 2007; Sutherland et al., 2003; Yoshida et al., 2005; Zheng et al., 2003; Pérez-Garijo et al., 2009).

Canton-S was used as WT, and the transgenic lines used are Gbe-Su(H)m8-lacZ (Furriols and Bray, 2001); P(UAS-Dl::NΔECN) (referred as *Nact*) (Baker and Schubiger, 1996; Doherty et al., 1996); P(UAS-tkv^Q199D^) (referred as *tkvA*), P(UAS-Dad.T) (Bangi and Wharton, 2006), MA33 (González-Reyes and St Johnston, 1998) and UAS-Dl.D (gift from M. Muskavith). Additional information can be found at FlyBase (http://flybase.bio.indiana.edu/).

Clones were generated by Flipase-mediated mitotic recombination on FRT19A, FRT40A, FRTG13, FRT2A, or FRT82B chromosomes (Xu and Rubin, 1993). To generate Dpp clones, the ywFLP; M(2L)24F ubiGFP FRT40A/CyO stock was used.

Ectopic expression of *tkvA*, *Dad* or *Nact* was performed by generating Flip-out Gal4 clones in animals carrying the hs-FLP22 and the A*y*GAL4 UAS-GFP transgenes (Ito et al., 1997). Flipase expression was induced by heat shocking 2-d-old females at 37.3°C for 1 h to generate mutant clones and Flip-out clones.

### Follicle staining and mounting

Ovaries from females were dissected directly into fixative 3 to 4 days after Flipase induction and stained following the protocol described in Grammont and Irvine (Grammont and Irvine, 2002). The following antibodies were used: goat anti β-galactosidase (1:1,000; Biogenesis), Mouse anti-Myc (1:100; Santa Cruz Biotechnology), rabbit anti-Myc (1:100; Santa Cruz Biotechnology, Inc.), mouse anti-GFP (1:500; Sigma-Aldrich), goat anti-GFP (1:1000; Abcam), rabbit anti-Zipper (1:1,000) (Jordan and Karess, 1997), mouse anti-Eya [1:500; Developmental Studies Hybridoma Bank (DSHB)], rat anti-ECad (1:200; DSHB), rat anti-NCad (1:20; DSHB), mouse anti-Notch (1:100; C458.2H, DSHB), mouse anti-Delta (1:200; C594.9B, DSHB), mouse anti-Dlg (1:50; DSHB), rabbit anti- phospho-Histone3 (PH3) (1:200; Cell Signaling), rabbit anti-PMad (1:200) (Nakao et al., 1997), rabbit anti-cleaved Caspase-3 (1:200; Cell Signaling), mouse anti-Hindsight (1:10; DSHB), Mouse anti-Phospho-Sqh (1:1000; Cell Signaling), mouse anti β-tubulin (1:1000; clone DM1A; Sigma-Aldrich), mouse anti-Armadillo (1:50; DSHB). Actin staining is realized by using Rhodamine-Phalloidin (Molecular Probes). Once stained, follicles were staged according to Spradling (Spradling, 1993). To avoid fluctuations of the depth of the follicles that are squeezed by the coverslip, each slide contains 15 ovaries, from which stage 11 to 14 are removed. After dissection of the follicles, most of the PBS is removed and 20 µl of the Imaging medium [PBS/Glycerol (25/75) (v/v)] is added before being covered by a 22/32 mm coverslip.

### Microscope image acquisition

Preparations were examined using a confocal microscope (LSM510 Meta; Carl Zeiss MicroImaging, Inc.) with 40× NA 1.3 plan-Neofluar and 63× NA 1.4 plan-Apochromat objectives. All imaging was performed at RT.

Images were processed using ImageJ (http://imagej.nih.gov/ij/). For all included images, a projection of all of the z sections in which the StC are visible is presented.

Image segmentation on early or mid stage 9 follicles was performed using an ImageJ macro (see supplementary material macro iMetrics and macro stack). Before running the macro, the stacks were cropped to exclude nurse cell membrane staining. Younger or older follicles were also removed and the canvas was adjusted to avoid follicles of interest touching the border of the image. Cell and follicle outlines are usually maximum intensity z projections of ∼2 µm. Non-specific background staining in the Eya channel was used to detect the outline of the follicles. For this, the AutoThreshold tool (Li dark method) was used to enhance contrast and the Analyse Particles tool is then applied to select large objects (superior to 2000 pixels). The angle of the object with respect to the x axis was calculated and printed. Extremities were obtained with the DrawFeretsTips function. Real Eya expression was then used to detect the anterior extremity: the standard deviation (SD) of pixel intensity was calculated at both extremities, where high SD indicates anterior. An Euclidian Distance Map was then applied (EDM16b function). To segment the cell outlines through the Ecad channel, the AutoThreshold tool (Median method – radius 10) was used to enhance contrast and the Analyse Particles tool was then applied to select objects (superior to 500 pixels). Mathematic morphometric tools (Close, Dilate And Erode) were then employed to close some of the outlines and then the Skeletonize tool was applied. Spikey outlines were smoothened with the PruneAll plugin. The Set Measurements was used to get the metrics of all the cell objects detected with the Analyse Particles (size = 50–1000) that have been registered in the roiManager.

**Fig. 2.**
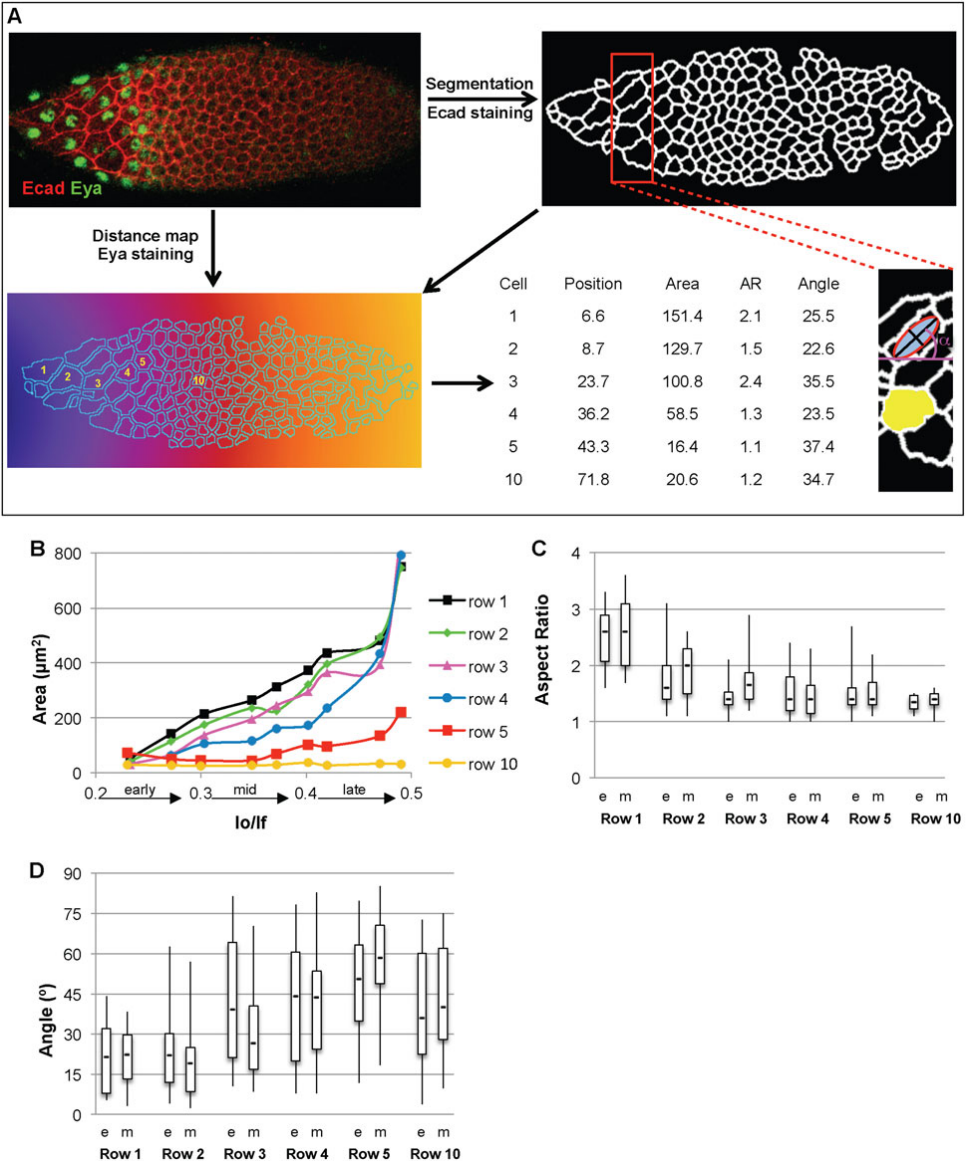
The physical parameters of StC flattening. (A) The custom-made ImageJ macro uses Ecad staining to segment the cells and Eya staining to extract follicle outlines, to determine the anterior and establish a distance map (for the stack used to describe the macro, see supplementary material macro stack). Six cells are numbered in function of the row they belong to. Apical surface area (Area in µm^2^), shape (Aspect Ratio), direction of the cell elongation (Angle in °) and position of the cells relative to the anterior (Mean Grey in µm) are given for each cell (see Materials and Methods). The red box in the segmented image is enlarged to show the Area (yellow) of one cell, the position of the best-fitted ellipse (red and grey) in another cell with the axes (black) used to calculate the Aspect Ratio and the Angle (purple). (B) Evolution of the apical surface area (Area) of WT StC per row during the stage 9 and early 10. The rows 1 to 4 contain cells that become StC. The row 5 contains cells that become either StC or main body follicular cells. The row 10 corresponds to a row of cells that become main body follicular cells. The follicles were staged by calculating the ratio between the length of oocyte (lo) and the follicle (lf). Each point represents average of more than 10 areas from 3 to 5 follicles at each lo/lf chosen. (C,D) Box and whisker plots of the shape (Aspect Ratio) (C) and of the direction of the cell elongation (Angle) (D) from WT early (e) and mid (m) follicles. Each sample represents at least 30 cells from 7 to 10 follicles. In all figures with box and whisker plots, boxes extend from to 25^th^ to 75^th^ percentile, with a line at the median. Whiskers extend to the most extreme values.

For each object, the Area is given in square units (µm^2^) and an ellipse is fitted. Information about the shape is obtained by measuring the primary (Major) and secondary (Minor) axis of the best fitting ellipse and by calculating the Aspect Ratio (AR) of the ellipse, where AR = [Major Axis]/[Minor Axis]. The direction of the cell elongation (Angle) is given by the angle formed by the primary axis and the X-axis of the image (Fig. 2).

Area, AR and direction of the cell elongation on old stage 9 or stage 10 follicles are obtained after the manual delimitation of the cell outlines based on a membrane-tagged GFP expression.

To calculate the volume of the follicles, we considered them as ellipsoid objects for which the volume is given by the formula: 3/4 π.a.b.c. a and b are the length and the width of the midsagittal plane image of the follicle. In normal follicles that are not under compression, c would be equal to b. Because we observe no significant increase in the length and width of our squeezed follicles in our mounting conditions, c is taken to be equal to b.

### Statistics

All the error bars shown in the figures are SD. p values are calculated using paired t test.

## RESULTS

### Determining the metrics of StC flattening

To clearly delineate stereotyped StC flattening, we created an ImageJ macro (called iMetrics) to semi-automatically measure a variety of cellular metrics. We monitor cell outlines using Ecad staining and these are used to get a skeleton of all cells and created a segmented image. Thereafter, apical surface area, cell shape and direction of the elongation are automatically extracted for each segmented cell (see Materials and Methods). In parallel, we used Eyes absent (Eya) expression, which turns on in the StC as they start flattening, to monitor not only the acquisition of the StC fate, but also the distance between these cells and the anterior end of the follicle. Based on this, StC are then sorted by M/L-oriented rows (Fig. 2A). To validate the functionality of the macro, we established these parameters for WT StC before, during and after stage 9. We found that the average apical surface area of StC between stage 8 and early stage 10A increases from 35±5 µm^2^ to 800±50 µm^2^, which corresponds to the net surface area of the StC growing from 0.2±0.2×10^4^ µm^2^ to 4.0±2.5×10^4^ µm^2^ (Fig. 2B). Our values are similar to those determined by Kolahi et al. (Kolahi et al., 2009). To complete these data, we carried out a row-by-row analysis of the dynamics of StC flattening during stage 9. We observed that 4 rows of cells are usually required to cover the nurse cell compartment (Fig. 2B). To test whether the macro was able to detect changes in cell shape and direction of the cell elongation, we analysed early and mid stage 9 follicles. At early stage 9, only StC in row 1 are significantly elongated (Aspect Ratio>2) and they are more elongated along the A/P axis than along the M/L axis (Angle α<30°). We observed that the StC in the row 2 are already oriented along the A/P axis, indicating that the anisotropy of the shape can be detected even before the cells are majorly elongated. By mid stage 9, the StC in rows 2 are elongated and the shape of the StC in row 3 is also more stretched (Fig. 2C,D). These data show that our tool accurately quantifies the magnitude and pattern of the flattening, and can follow the dynamics of each row.

### The TGFβ pathway is required for the dynamic and the degree of flattening

**Fig. 3.**
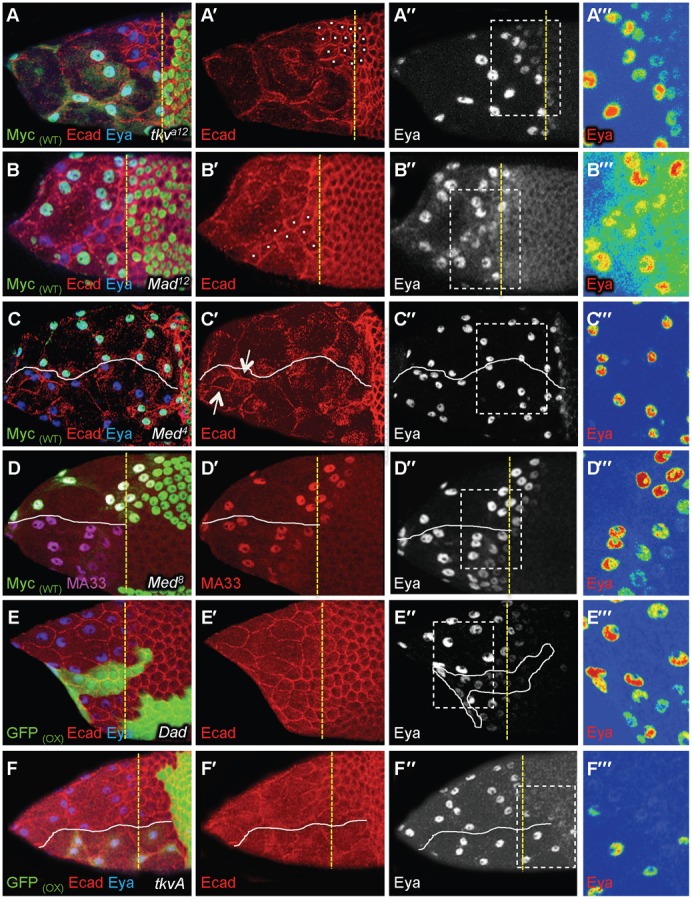
TGFβ is required for cuboidal-to-squamous transition. In all figures, the yellow dotted line marks the A/P position of WT flattening StC and the white line separate the mutant cells from WT cells; the Myc_(WT)_ and GFP_(WT)_ labels indicate that WT cells are green whereas the GFP_(OX)_ label indicates that cells overexpressing a transgene are green; the use of a colour gradient allows the visualization of differences in intensities of protein accumulation from none (blue) to strongest (red); A‴–F‴ are magnified views of the boxes drawn in A″–F″. (A–F‴) Stage 9 (A,B,D–F) or 10 (C) follicles with clones of mutant cells (A–D) or with clones of cells over-expressing Dad (E) or *tkvA* (F). A‴–F‴ are magnified views of the boxes drawn in A″–F″. (A,B) Difference of dynamic of AJ remodelling between WT StC and mutant StC (white dot) in round or drawn-out clones. Mutant StC undergoing flattening were located three to four rows anterior to WT StC. (C) Follicle with *Med* clones. The arrows point to persistent AJ within clones. (D) Comparison of MA33 expression between *Med* and WT StC. (E) Difference of AJ remodelling dynamic between Dad and WT StC (n = 59). (F) Difference of dynamic of the flattening between *tkvA* and WT StC. In (C–F) a white line separates mutant, Dad or *tkvA* StC or from WT StC. All the phenotypes have been observed with different alleles of *tkv*, *punt*, *Mad* and *Med*: for *tkv* (*tkv^a12^*: n = 50; *tkv^8^*: n = 88, *tkv^7^*: n = 42); for *punt* (*punt^Δ61^*: n = 30; *punt^135^*: n = 20; *punt^1046^*: n = 18), for *Mad* (*Mad^1-2^*: n = 90, *Mad^12^*: n = 40, *Mad^8-2^*: n = 35), and for *Med* (*Med^8^*: n = 210; *Med^13^*: n = 123, *Med^4^*: n = 134).

**Fig. 4.**
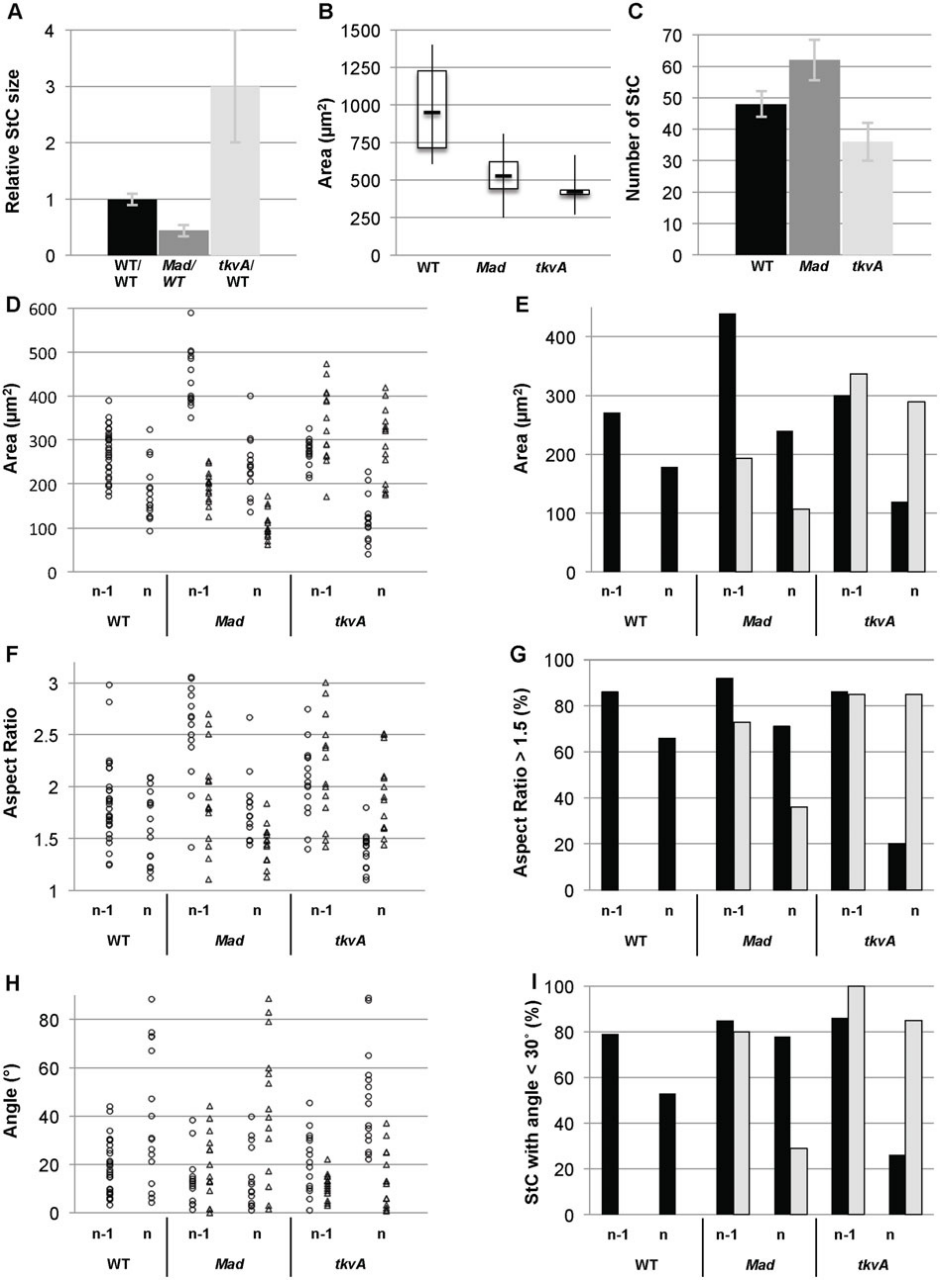
TGFβ controls the timing, the degree and the dynamic of StC flattening. *Mad* follicles refer to follicles containing 40 to 60% of *Mad^12^ or Mad^1-2^* StC. Note that no measurement has been done on follicles containing 100% of mutant StC, as such follicles die prematurely. *tkvA* follicles refer to follicles containing more than 50% (A,D–I) or 80% (B,C) of *tkvA* StC. (A) Relative size of WT, *Mad* or *tkvA* StC during flattening. Ratios have been calculated by measuring Area of WT, *Mad* or *tkvA* StC located in the same row than WT flattening StC in WT, *Mad* or *tkvA* follicles, respectively. Each sample represents at least 28 cells from stage 9 follicles (n = 5) (0.3<lo/lf>0.35). (B) Box and whisker plots of apical surface area (Area) from WT, Mad and *tkvA* follicles. Each sample represents at least 35 cells from stage 10 follicles (n = 10) (lo/lf = 0,5). (C) Determination of the number of StC in WT (n = 10), *Mad* (n = 18) or *tkvA* (n = 22) follicles (lo/lf = 0.5). (D,F,H) Apical surface area (Area), shape (Aspect Ratio) or direction of the cell elongation (Angle) of individual StC is plotted in function of the row: flattening (row n) and flattened (row n-1); and of the genotype: WT (dots), *Mad* (triangles) or *tkvA* (triangles). Each sample represents 9 to 14 cells from 4 to 5 follicles (lo/lf = 0.35). (E) Average of apical surface area (Area) of StC per row and genotype: WT (black bar), Mad (grey bar) or *tkvA* (grey bar). (G) Percentage of StC displaying an Aspect Ratio superior of 1.5, per row and genotype. (I) Percentage of StC elongated in a direction making an angle superior of 30° with the A/P axis, per row and genotype. (E,G,I) Each sample represents 15 to 30 cells from 10 follicles.

TGFβ signalling activity has previously been detected during the cuboidal-to-squamous follicular cell transition (Guichet et al., 2001; Muzzopappa and Wappner, 2005; Twombly et al., 1996), but it is unclear if it is required for this process. To test this, we examined Ecad and Eya expression patterns in follicles carrying somatic mutant clones for all canonical TGFβ signalling components. The robustness of the phenotypes was assayed by using three different mutant alleles for each gene. No phenotype was detected before stage 9 in any follicular cell (supplementary material Fig. S1A,B) or during stage 9 when the mutant clone encompassed only the main body follicular cells (supplementary material Fig. S1C,D). In contrast, more than 80% of the stage 9 follicles with StC mutant for either *tkv*, *punt*, *Mad* or *Med* or with StC over-expressing the negative regulator Dad displayed an aberrant cuboidal-to-squamous transition, based on the four following observations. First, mutant StC presented a delay in AJ remodelling compare to WT StC, as perdurance of apical membrane-localized Ecad molecules is observed in mutant StC, indicating that TGFβ signalling is required for temporal AJ remodelling in these cells (Fig. 3A,B,C; supplementary material Fig. S1E). Second, mutant StC showed a delayed onset of Eya and of the MA33 enhancer-trap StC marker expression, indicating that TGFβ signalling is required for the expression of both markers (Fig. 3A,B,D,E). Third, we quantified the degree of flattening of WT and mutant StC in mosaic follicles containing 40% to 60% mutant StC. At mid stage 9, mutant StC that are flattening or have already flattened are on average half the size of WT StC (Fig. 4A,D,E). At early stage 10, the apical surface area of the mutant StC is reduced by 45% (Fig. 4B) while the volume of the mosaic follicles stays similar to that of WT follicles (supplementary material Fig. S1F), indicating that TGFβ signalling controls the degree of flattening. Fourth, we counted 25% extra Eya or MA33-expressing cells over the nurse cell compartment in mutant stage 10 follicle (Fig. 4C). This increase in StC number could be due to either main body follicular cells that have been recruited and converted to StC or from extra rounds of cell division. We excluded the latter possibility because we never detected phospho-Histone3 expression in mosaic *Med* follicles after stage 6 (supplementary material Fig. S1G). Thus, the extra cells over the nurse cells in mosaic follicles are main body follicular cells that have adopted a StC fate. Together, these data demonstrate that the TGFβ pathway is required for the cuboidal-to-squamous transition.

We also analysed the shape and the direction of the elongation of the mutant StC. We observed that the mutant StC elongated later and less than WT cells (Fig. 4F,G), but along the A/P axis (Fig. 4H,I). Our measurements also show that the improper flattening of mutant StC has an impact on the flattening of neighbouring WT StC. Indeed, the apical surface area of WT StC in mosaic stage 9 follicles is wider than those of StC in WT follicles. A higher proportion of the former also more rapidly adopts an elongated shape oriented along the A/P axis than the latter (Fig. 4D–I). This confirms that proper StC flattening requires coordination between adjacent cells. The expressivity of the phenotypes also depends on the size (fewer than 5 cells versus more than 5 cells) and on the shape of the mutant clones (round versus drawn-out) (compare Fig. 3A with Fig. 3B). Isolated mutant cells do not flatten at all and maintain AJ until stage 10. These data show that TGFβ pathway controls the dynamics of the cuboidal-to-squamous transition, the degree of flattening of the StC and participates to the development of an elongated cell shape.

Only 50% of follicles with somatic *sax^4^* clones display a mild phenotype (compare supplementary material Fig. S1H with Fig. 3A,B). We also tested whether the expression of the ligands *dpp* and *gbb* ligands in the stretched cells is required for cell flattening by generated either RNAi-expressing clones and or clones for hypomorphic or null alleles. We observed that follicles where *dpp* RNAi clones encompass most of the StC, present smaller and supernumerary StC compare to WT follicles. In addition, these StC do not remodel their AJ. However, although some mutant clones (33%) that encompass less that 15 StC, contain cells that are smaller that their WT neighbours, most clones do not induce a phenotype, indicating that the Dpp ligand expressed by the WT cells rescues the *dpp* StC (supplementary material Fig. S1I,J). Clones for hypomorphic *dpp* alleles in a Minute background, which helps recover some mutant cells, displayed phenotypes that were too variable to be conclusive (data not shown). Lastly, follicles with somatic *gbb^1^* clones do not display any abnormal cell flattening. Thus, Dpp is the main ligand that is used to drive cell flattening and its expression in the StC is required for the flattening. No detectable phenotypes were observed in follicles with *wit^G15^* clones, or in follicles with somatic clones of two members of the Activin pathway, *baboon* or *Smad* on *X* (supplementary material Fig. S2A–D).

### The TGFβ pathway controls the timing of StC flattening

To test whether the TGFβ signalling activity is sufficient to promote the cuboidal-to-squamous follicular cell transition, we expressed a constitutively active form of *tkv* (*tkvA*) (Lecuit et al., 1996) (Nellen et al., 1996) using the Flip-out technique. During stage 9, when follicles are composed of 50% *tkvA*-expressing StC (referred to as *tkvA* StC), these StC exhibit premature AJ remodelling and precocious Eya expression. During the cuboidal-to-squamous transition, *tkvA* StC undergoing flattening also present a three fold increase of their apical surface when compared to those of WT neighbours (Fig. 4A). This increase is temporary, as, in a stage 10, the apical surface area of flattened *tkvA* StC (844±45 µm^2^) is not significantly different from that of flattened WT StC (A = 750±30 µm^2^) (p>0.05) (Fig. 3F and Fig. 4D,E). Interestingly, the *tkvA* StC flatten progressively from the anterior to the posterior, indicating that the A/P gradient of flattening is independent of the spatio-temporal pattern of TGFβ activity (supplementary material Fig. S3A). *tkvA* StC also take on a more elongated shape oriented along the A/P axis than WT cells (Fig. 4F–I). In addition, the increased flattening of the *tkvA* StC also has an effect on the shape of their WT counterparts, since only 20% of these are elongated (Aspect Ratio>1.5), as compared to the 65% for WT flattening StC in WT follicles. However, this non cell-autonomous effect is temporary, as no difference is detected once they are fully flattened. We also analysed follicles composed of at least 80% *tkvA* StC. Based on the overall size of such follicles, flattening starts at stage 8 and ends by mid-stage 9 (lo = 0.5lf) (supplementary material Fig. S1F). At this point, the apical surface area of *tkvA* StC is smaller by 2.25-fold compared to that of StC from stage 10 WT follicles and the number of StC is reduced by 25% compare to WT follicles (Fig. 4A,C). We hypothesized that this could simply be a consequence of the reduced size of such follicles (supplementary material Fig. S1F). To confirm this, we compared the apical surface area of the *tkvA* StC with those of WT StC from WT follicles of similar size and we observed that *tkvA* StC are neither smaller nor wider than WT StC (supplementary material Fig. S3B). In parallel, we also verified that the low number of StC is not due to cell death since cleaved-Caspase3 expression is never detected (supplementary material Fig. S3A). Thus, *tkvA* StC flatten to the same extent as WT cells and adjust their degree of flattening and their number to the size of the nurse cell compartment. Together, these data demonstrate that the premature activation of TGFβ signalling is sufficient to induce cell flattening in anterior cells and modifies the dynamics of the flattening in an opposite manner to the lack of signalling. But, it does not affect the other four main features of flattening, namely the A/P gradient, the degree of flattening, the shape of the StC and the direction of the cell elongation. From these data, we confirm that the activation of the TGFβ pathway controls the dynamics of StC flattening and we also demonstrate that the activation of the TGFβ pathway controls the timing of this process.

We further examined whether the ectopic activation of TGFβ signalling is sufficient to promote premature cuboidal-to-squamous transition. When TGFβ was activated only in the main body follicular cells or in the follicular cells between stage 2 and 6, they did not present AJ disassembly and did not flatten based on the expression of a lateral and basal marker, Disc large, but did show a 2-fold increase in their apical area when compared to WT cells (supplementary material Fig. S3C–F). Thus, ectopic induction of TGFβ signalling induces cell growth, but not a cuboidal-to-squamous transition.

### TGFβ signal controls Notch and Delta expression patterns

**Fig. 5.**
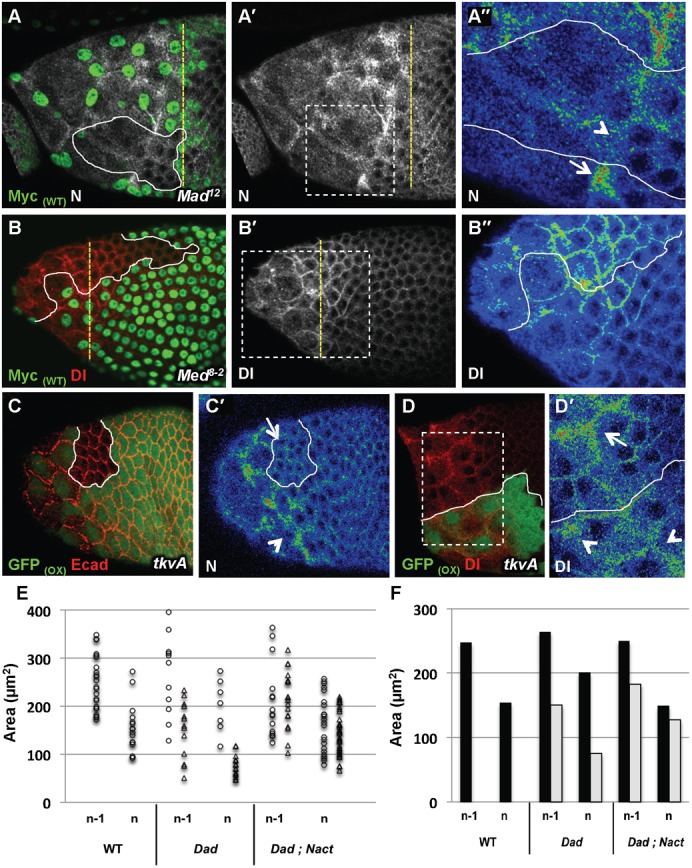
TGFβ controls N and Dl expression patterns. (A–B″) Stage 9 follicles with clones of *Mad* or *Med* cells (A B). A″,B″,D′ are magnified views of the boxes drawn in A′,B′ and D. (A) N expression at AJ undergoing remodelling in *Mad* (arrowhead) or WT StC (arrow) (n = 10 for *Mad* and n = 8 for *Med*). (B) Dl expression in *Mad* or in WT StC. (n = 8 for *Mad* and n = 7 for *Med*) (C,D) Follicles with clones of cells over-expressing *tkvA*. (C,C′) N expression at the AJ undergoing remodelling of *tkvA* StC (arrowhead) or of WT cells (arrow) in a stage 8 follicle (n = 23). (D,D′) Dl expression in *tkvA* StC (arrowhead) or in WT StC (arrow) at stage 9 (n = 19). (E) Apical surface of area (Area) of individual StC is plotted in function of the row: flattening (row n) and flattened (row n-1); and of the genotype: WT (dots), UAS-Dad (triangles) or UAS-Dad; UAS-Nact (triangles). Each sample represents 9 to 14 cells from 3 to 5 follicles (lo/lf = 0.35). (F) Average of apical surface area (Area) of StC per row and genotype: WT (black bar), UAS-Dad (grey bar) or UAS-Dad; UAS-Nact (grey bar). Each sample represents 15 to 30 cells from 10 follicles.

Three genes or signalling pathways, Hindsight, Tao and the Notch pathway, are known to be required for StC flattening. We observed no differences in Hnt expression in *Mad* clones, indicating that Hnt acts in parallel to TGFβ signalling for StC flattening (supplementary material Fig. S4A). We did not test whether TGFβ controls Tao, because Tao acts before the pathway is active (Gomez et al., 2012). For the Notch pathway, we previously described that N and Dl are strongly detected at the membranes of the StC that just flattened and of the StC that are flattening (supplementary material Fig. S4B,C) (Grammont, 2007). At these membrane contacts, we observed a strong reduction of N in *Mad* or *Med* clones (Fig. 5A). In contrast, a strong increase of Dl is detected in the *Mad* or Med StC undergoing flattening and up to three/four rows posterior to these cells (Fig. 5B), which clearly differs from WT expression. We then used the *GbeSu(H)-lacZ* transgene to determine whether the pathway is affected, and we detected a delayed and reduced expression of N reporter activity in *Mad* clones (supplementary material Fig. S4D), indicating that TGFβ signalling is required for proper N activity in the flattening StC. To test whether TGFβ signalling is sufficient to induce N expression and to repress Dl expression, we analysed N and Dl expression patterns in *tkvA* cells. At stage 8, N is detected in the *tkvA* StC, showing that TGFβ signalling controls the temporal pattern of N expression (Fig. 5C). However, TGFβ signalling does not induce N expression in the main body follicular cells, indicating that the spatial pattern of N is independent of TGFβ. Dl expression is lower in *tkvA* cells than in WT cells before and during StC flattening (Fig. 5D; supplementary material Fig. S4E), indicating that TGFβ signalling can repress Dl expression at several stages of oogenesis. Nevertheless, this repression is insufficient to fully shut down Dl expression, as it is still present in *tkvA* StC. Thus, Dl is controlled by at least one other pathway, which overcomes the negative regulation exerted by TGFβ signalling. From these results, we hypothesized that the opposite regulation of TGFβ on N and Dl could be important to prevent them from being expressed simultaneously in the same cell, a context that is known to prevent Notch activation through cis-interactions (de Celis and Bray, 1997; Jacobsen et al., 1998; Klein et al., 1997; Micchelli et al., 1997). To support this hypothesis, we over-expressed Dl and observed that StC flattening is impaired in Dl-expressing cells as well as in neighbouring WT cells, showing that Dl expression must be regulated sequentially to allow proper StC flattening (supplementary material Fig. S4F).

To test whether the phenotype observed in the absence of TGFβ signalling could derive entirely from an impaired N signalling, we measured the apical surface of flattened and flattening StC in stage 9 follicles composed of WT cells and of cells expressing both Dad and a constitutive active form of N (Nact) and compared it with the apical surface of StC from WT follicles and from follicles expressing Dad alone (compare Fig. 3E with supplementary material Fig. S4G). At mid stage 9, although the *Dad* StC are around half the size of WT StC, the StC overexpressing concomitantly Dad and Nact display a wider apical surface than those of the Dad StC (Fig. 5E,F), indicating that Nact expression can rescue partially the phenotypes associated with impaired TGFβ signalling (p<0.005). Taken together, these results demonstrate that TGFβ activity orchestrates cell flattening by triggering the N pathway and that, once this pathway is activated, either of the pathway regulate common targets and/or Notch fulfils some of the TGFβ requirements.

### TGFβ signalling regulates Actomyosin contractibility and Ncad down-regulation

**Fig. 6.**
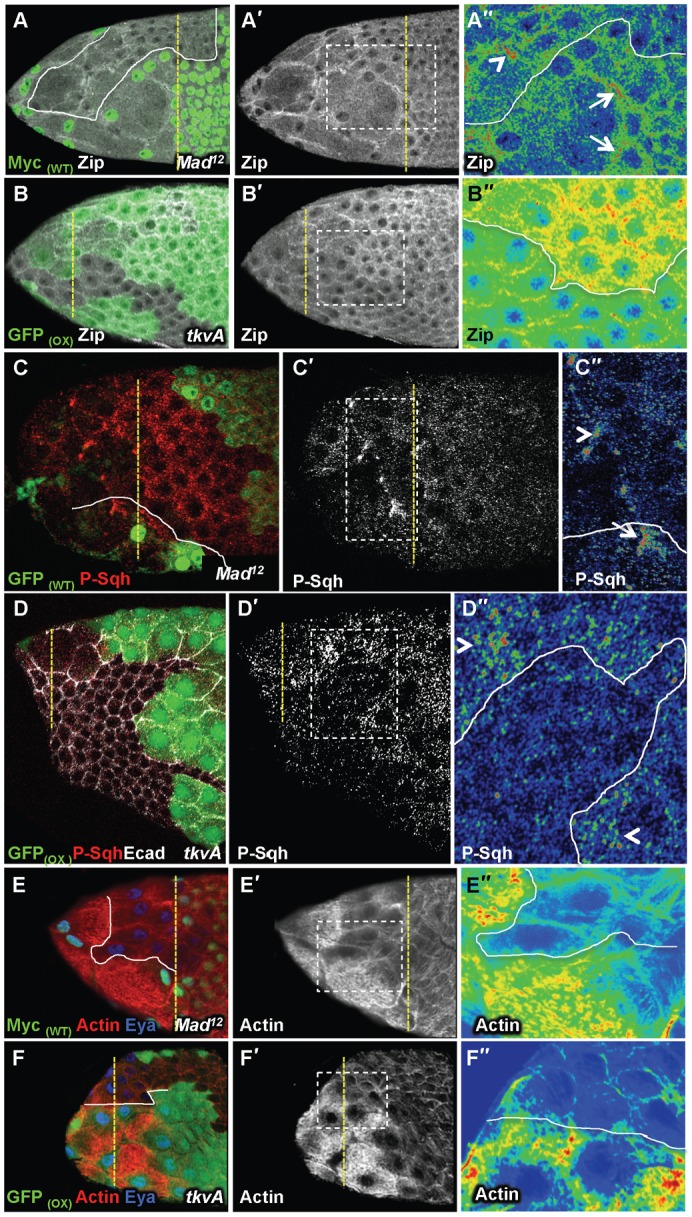
TGFβ controls Actomyosin network formation and contractibility. (A–F″) Stage 9 follicles. A″–F″ are magnified views of the boxes drawn in A′–F′. Zip and P-Sqh accumulations and Actin network in *Mad* (n = 40 for Zip, n = 16 for P-Sqh, n = 40 for Actin) (A,C,E) or *tkvA* (n = 80 for Zip, n = 22 for P-Sqh and n = 40 for Actin) (B,D,F) follicles. The point at the Anterior AJ of StC undergoing flattening are marked with arrows in WT and with arrowheads in *Mad* or *tkvA* clones.

As the main target that Notch controls is the localisation of the Myosin II components (Grammont, 2007), we tested whether impaired TGFβ signalling also leads to abnormal activity of the Actomyosin network at AJ undergoing disassembly. We analysed the levels of expression of the heavy chain of Myosin II (Zip), of the phosphorylated and active form of the regulatory light chain (P-Sqh) and of F-actin in follicles carrying StC that were either mutant for the TGFβ pathway or expressing *tkvA* during stage 9. In WT cells, Zip and Sqh accumulate at the junctions that undergo disassembly (Grammont, 2007). We observed that accumulation of both components is delayed in *Mad* or *Med* StC (Fig. 6A,C; data not shown). Consistent with this, a high and uniform level of Zip expression is detected in all *tkvA* cells and a premature accumulation of P-Sqh is observed in *tkvA* StC undergoing flattening (Fig. 6B,D). For the Actin filament network, we observed the density of Actin filaments increases in WT StC undergoing flattening and remains high in flattened cells. This does not occur in *Mad* StC, whereas it does occur earlier in *tkvA* StC (Fig. 6E,F). This *tkvA*-dependent effect is specific of its expression in the StC as no defect was observed in the main body follicular cells (Fig. 6F; supplementary material Fig. S5A). The TGFβ signal is thus required to promote the formation of actin bundles and to control the activity of Myosin II in WT follicles.

DN-cadherin (Ncad) down-regulation is another step that needs to be regulated to allow StC flattening. Indeed, we previously showed that Ncad expression decreases from stage 7 in WT follicles, and that the maintenance of this expression blocks StC flattening (Grammont, 2007; Tanentzapf et al., 2000; Zartman et al., 2009). In 100% of *Mad* or *Med* mutant clones, Ncad is detected in anterior cells at stages 8 and 9 (supplementary material Fig. S5B), indicating that the TGFβ pathway is required for down-regulating Ncad expression. To confirm this, we activated the TGFβ pathway between stages 4 and 7, and observed that Ncad expression is greatly reduced or absent in 100% of *tkvA* follicles (supplementary material Fig. S5C). This result confirms that the temporal control of TGFβ activity throughout oogenesis is crucial in order to achieve the proper expression pattern of this adhesion molecule. Thus, TGFβ signalling orchestrates StC flattening via multiple regulatory inputs of adhesion molecules and of the components of the actin cytoskeleton.

As TGFβ is regulating N activity, we also tested whether Notch could be also involved in *Ncad* regulation. In stage 9 follicles with *Dl* clones, we observe the persistence of Ncad expression, indicating that *Ncad* represents a second shared target for TGFβ and Notch signalling (supplementary material Fig. S5D).

## DISCUSSION

During stage 9 of oogenesis, the follicular epithelium exhibits morphogenetic rearrangements, including a cuboidal-to-squamous transition at the anterior pole of the follicle. Here, we investigate the role of TGFβ signalling in this transition by first collecting detailed cellular metrics in WT and mutant conditions. We then use these metrics to generate databases in order to enable the quantitative phenotypic characterisation of various mutants, of interactions between these mutants, and of the extent of rescue by the forced expression of a gene in different mutant contexts. From these analyses on TGFβ signalling, we conclude that the TGFβ pathway: (i) is essential in controlling the timing, the dynamics and the degree of flattening; (ii) plays a role in determining the elongated, final shape of the StC, and (iii) does not control the direction of cell elongation or the A/P-oriented wave of flattening. We show that StC flattening is dependant on *dpp* expression in the StC and that the activation of the pathway occurs in an autocrine and paracrine manner, as WT cells can locally rescue cells impaired for Dpp production. As no phenotype is observed in absence of Gbb, Dpp homodimers are likely to be used to activate the pathway, rather than Dpp/Gbb heterodimers. All the other canonical TGFβ components are necessary for StC flattening and their absence leads to severe defects of flattening, except for *sax*. Indeed, the contribution of *sax* to StC flattening is minor, when compared to that of *tkv*, which is in accordance with Sax being the primary receptor for Gbb signalling and with our data showing that *gbb* is not required (Bangi and Wharton, 2006; Dorfman and Shilo, 2001; Singer et al., 1997). Thus, TGFβ must signal principally through Tkv and Punt in the StC. Importantly, our data show that progressive TGFβ activation along the A/P axis in WT follicles is irrelevant for the A/P-oriented flattening and suggests instead that the cue(s) responsible of this feature itself could activate TGFβ signalling in a wave starting at the anterior tip of the follicle. In addition, the propagation of TGFβ from anterior to posterior could be helped by the extra-cellular matrix, given that follicles are surrounded by a Collagen IV-enriched basement membrane. Indeed, recent data implicate Collagen IV in regulating the distribution of TGFβ in the germarium and in the embryo (Wang et al., 2008). However, although it has been shown that the basal membrane is involved in shaping the follicle and that the shape of the follicle may in turn impact StC flattening (Haigo and Bilder, 2011), there is currently no direct evidence implicating any components of the basement membrane in this process. Elucidating the role of the basement membrane and its attendant physical characteristics on StC flattening could thus represent an exciting new avenue of research.

The transformation from a cuboidal to a squamous epithelium involves shrinking the lateral membrane as well as extending the basal and apical membranes. Shrinking the lateral membrane requires an adjustment of cell adhesion properties; in this regard, it has been shown that the reduction of Fas2 and the remodelling of AJ are required for the lateral shrinking of the StC (Gomez et al., 2012; Grammont, 2007). Our analyses reveal that TGFβ signalling is responsible for AJ disassembly, for increasing actin filament formation and its local contractibility through the localization of the non-muscular Myosin II at the AJ undergoing remodelling, and for turning off Ncad. Thus, TGFβ generates cell-autonomously internal forces that likely participate to remodel AJ and lead to the shrinkage of the lateral side of StC.

Until recently, the mechanism that leads to cell flattening in *Drosophila* follicles have remained unstudied, possibly because follicular cell stretching has long been considered as a passive event in response to germline growth. This possibility has been reinforced by the measurement of the compliance of the StC before, during and after the flattening, and by the analysis of *dic* mutant follicle, in which the germline growth is reduced (Kolahi et al., 2009). If StC were entirely passive during this process, it should be impossible to modulate this process, by manipulating gene expression in the soma, except if the genes encoded for direct cell shape regulators, such as Myosin, Actin, adhesion molecules etc. However, our data show first the TGFβ signalling pathway is cell-autonomously required for StC flattening. Second, this pathway modulates expression and activity of direct cell shape regulators, which are known to produce internal forces. Third, StC flattening occurs prematurely when TGFβ pathway is activated in the soma at young stages, demonstrating that providing TGFβ is sufficient for the cells to flatten and that StC do not just flatten through a compliant process. Altogether, our data reveal that StC must to be instructed by TGFβ in order to respond to the global force generated by the germline growth and bring out the importance of local forces in shaping and flattening the StC.

Besides its role in regulating several cell shape cell effectors (Ecad, Ncad, Actin and Myosin), we also demonstrate that TGFβ is essential for Notch expression. Our current and past results show that they are both required for the accumulation of the non-muscular Myosin II at the AJ that need to be remodelled, and for the down-regulation of Ncad. Whether TFGβ acts on these targets entirely via its control of the Notch pathway or whether they both control common targets still need to be established. Importantly, we also observe that TGFβ signalling negatively regulates Delta in the cells posterior to the row of StC undergoing flattening and that proper StC requires sequential Dl expression. Finding the mechanisms that protects Dl expression from the negative input exerted by TGFβ signalling would help understanding the mechanism driving the sequential activation of Notch and the progression of the A/P wave of flattening.

The process of AJ remodelling may resemble, in part, some processes observed in the epithelial-to-mesenchymal transition (EMT) (Zavadil and Böttinger, 2005). Indeed, EMT is characterized by remodelling of epithelial cell-cell and cell-matrix adhesion contacts and of their actin cytoskeleton, by losing epithelial polarity and by acquiring the capacity for individual motility. Although it is still unclear whether cell polarity is affected during StC flattening and to what extent cell-matrix contact are remodelled, StC flattening still shares two key characteristics with EMT: AJ disassembly and actomyosin remodelling. Interestingly, TGFβ is known to be a strong inducer of EMT (Miettinen et al., 1994) during developmental or oncogenic events, leading to the hypothesis that some of the processes observed during StC flattening could be controlled in a similar way to those observed during TGFβ-dependent EMT.

## Supplementary Material

Supplementary Material
