# Supplementary material for: Transforming Growth Factor β/activin signalling induces epithelial cell flattening during Drosophila oogenesis
Source: Biol Open. 2015 Feb 13;4(3):345–54. doi: 10.1242/bio.201410785 (PMC4359740; doi:10.1242/bio.201410785)
Supplement: Supplementary Material [file supp_4_3_345__index.html]

Transforming Growth Factor β/activin signalling induces epithelial cell flattening during Drosophila oogenesis — Supplementary Material 

# Transforming Growth Factor β/activin signalling induces epithelial cell flattening during *Drosophila* oogenesis

## bio.201410785 Supplementary Material

**Files in this Data Supplement:**

- Supplementary Material - Isabelle Brigaud et al. doi: 10.1242/bio.201410785
- Macro iMetrics
- Macro stack - The stack used to describe the macro in Fig 2.
