# Supplementary material for: Transforming Growth Factor β/activin signalling induces epithelial cell flattening during Drosophila oogenesis
Source: Biol Open. 2015 Feb 13;4(3):345–54. doi: 10.1242/bio.201410785 (PMC4359740; doi:10.1242/bio.201410785)
Supplement: Supplementary Material [file supp_bio.201410785_bio.201410785-s1.pdf]

Supplementary Material

Isabelle Brigaud et al. doi: 10.1242/bio.201410785

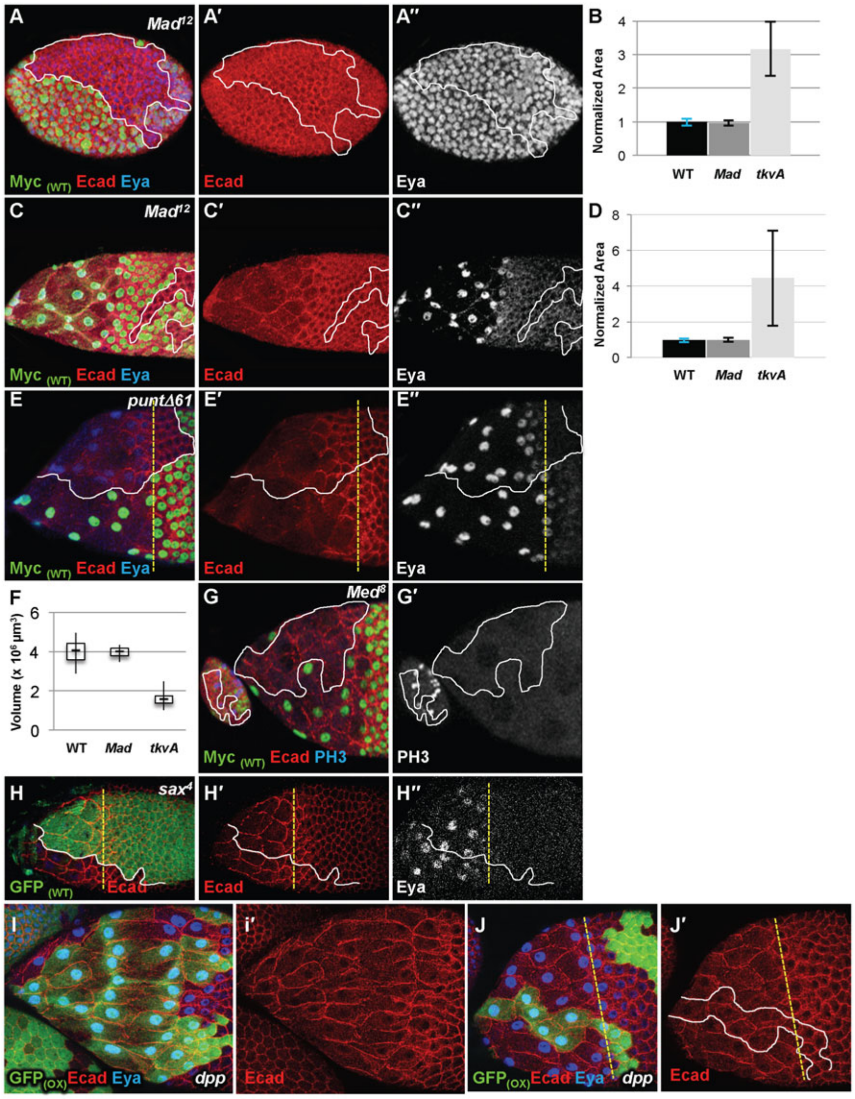

Fig. S1. TGFβ is required at stage 9 in the StC. The white line separates mutant from WT cells. (A–A″) Stage 6 follicle with *Mad* clones (n=35). (B,D) Normalized apical surface area (Area) of the WT, *Mad* and *tkvA*-expressing cells at stage 7 (B) and at stage 9 (D). For each sample, at least 50 cells have been measured from 3 to 5 follicles. Values were normalized by calculating a ratio with the average size of WT cells. (C–C″) Stage 9 follicle with *Mad* clones encompassing only the main body follicular cells (n=52). (E–E″) Stage 9 follicle with *punt* clones. (F) Box and whisker plot of volume of the WT, *Mad* and *tkvA*-expressing follicles. *Mad* follicles refer to follicles containing 40 to 60% of *Mad* StC. *tkvA*-expressing follicles refer to follicles containing more than 80% of *tkvA*-expressing StC. Each sample represents at least 25 stage 10A follicles (lo=0.5lf). (G–G′) Stage 5 and 9 follicles with *Med* clones stained with phospho-Histone3 antibody (*Med^8*: n=35). (H–H″) Stage 9 follicle with *sax* clones (n=82). (I–J′) Stage 9 follicles with clones expressing RNAi against *dpp* (n=31).

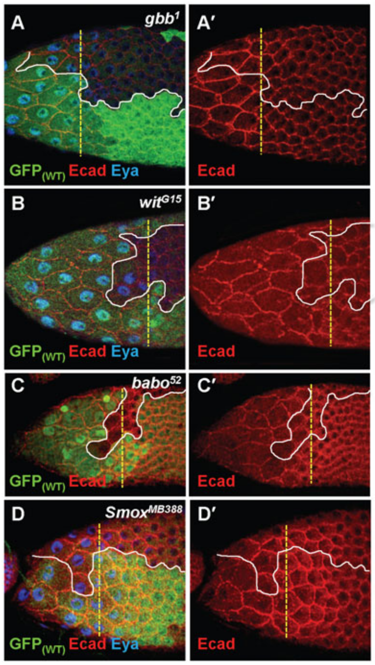

Fig. S2. Some components of the TGFβ or Activin pathway are not required for StC flattening. (A–D′) Stage 9 follicles with mutant clones for *gbb* (n=70), *wit* (n=19), *babo* (n=20) and *Smox* (n=20).

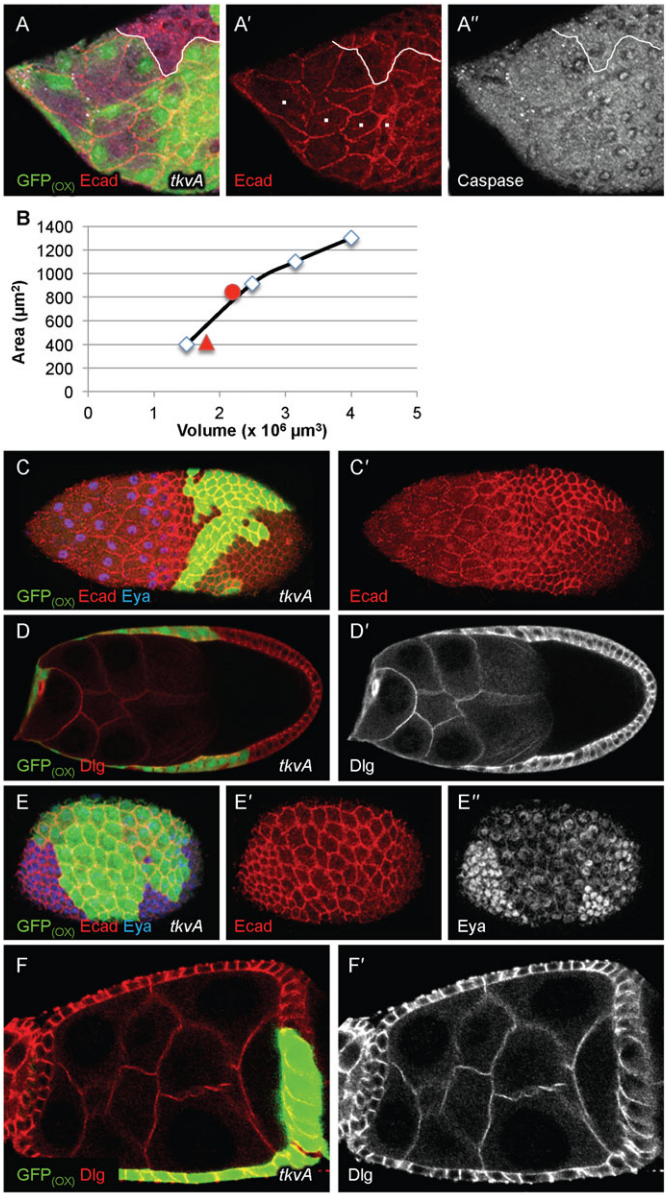

Fig. S3. Precocious *tkvA* expression induces cell growth. (A–A″, C–F′) Follicles with clones expressing *tkvA*. (A) Caspase expression in follicle with most of the StC expressing *tkvA* (n=20). The A/P gradient of flattening is visible (dots). (B) Evolution of the apical surface area (Area) of the WT StC in function of the volume of WT follicles (white diamond). The red triangle corresponds to the average of apical surface of StC in follicles (lo/lf=0.5) containing more than 80% of the StC expressing *tkvA* (n=7). The red circle corresponds to the average of apical surface area of StC in follicles (lo/lf=0.5) containing 50% of the StC expressing *tkvA* (n=7), for which no change in volume is detected. (C) Influence of the over-expression of *tkvA* in the main body follicular cells at stage 9 (n=50). (D) Expression of Dlg in the StC and main body follicular cells at stage 9. (E) Influence of the over-expression of *tkvA* in the cells at stage 6 (n=35). (F) Expression of Dlg at stage 5.

Biology Open (2015) 000, 1–10 doi:10.1242/bio.201410785

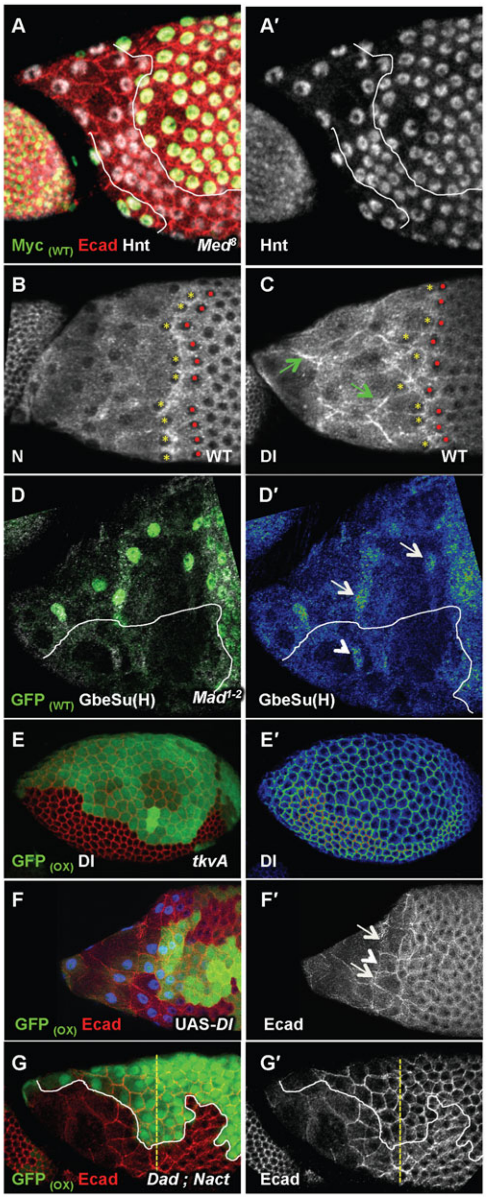

Fig. S4. TGFβ signalling controls N activity. (A,A′) Hnt expression in a stage 9 follicle with *Med* clones. (B,C) N (B) and Dl (C) expression in WT StC at the membrane contacts between the StC that just flattened (yellow star) and the StC that are flattening (red dot). In C, arrows (green) point to Dl expression in flattened StC. (D,D′) Expression of the *GbeSu(H)-lacZ* transgene in *Mad* StC (arrowhead) and WT StC (arrow) at stage 9 (n=12). (E,E′) Dl expression in stage 7 follicle with clones over-expressing *tkvA*. (F,F′) Over-expression of Dl in a stage 9 follicle (n=19). Asterisks (green) label Dl-expressing cells. (G,G′) AJ remodelling in stage 9 follicles with clones over-expressing *Dad* and *Nact* (n=34).

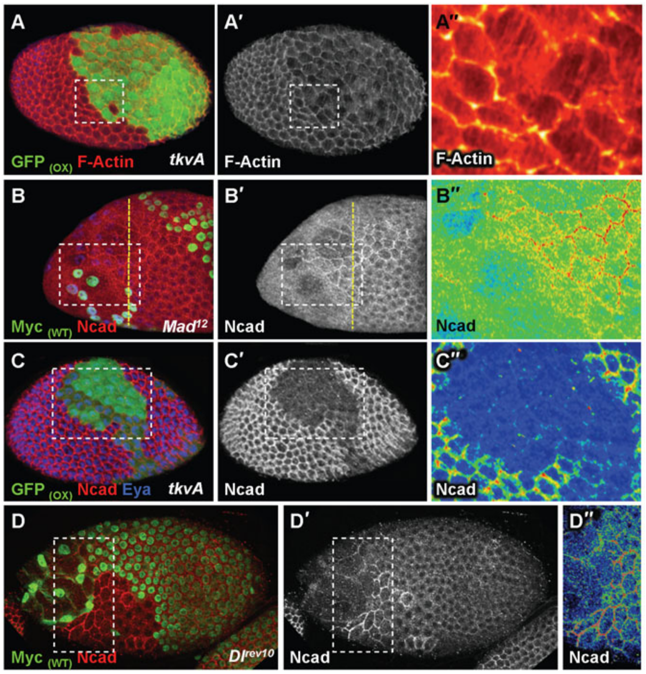

Fig. S5. TGFβ signalling controls Ncad downregulation. A″,B″,C″,D″ are magnified views of the boxes drawn in A,A′, B,B′, C,C′ and D,D′, respectively. (A–A″) Basal expression of F-Actin in *tkvA* cells in a stage 7 follicle. (B–C″) Ncad expression in *Mad* mutant clones at stage 9 (n=10 for *Mad* and n=20 for *Med*) (B) or in *tkvA* cells at stage 6 (n=20) (C). (D–D″) Ncad expression in *Dl* clone in a stage 9 follicle (n=9).

Supplementary material macro iMetrics and macro stack: See supplementary webpage
