# Supplementary material for: Transforming Growth Factor β/activin signalling induces epithelial cell flattening during Drosophila oogenesis
Source: Biol Open. 2015 Feb 13;4(3):345–54. doi: 10.1242/bio.201410785 (PMC4359740; doi:10.1242/bio.201410785)
Supplement: Supplementary Material [file supp_bio.201410785_Macro_iMetrics.docx]

**Transforming Growth Factor ß/activin signalling induces epithelial cell flattening during *Drosophila* oogenesis**

Isabelle Brigaud^1^, Jean-Luc Duteyrat^1^, Julien Chlasta^1,2^, Sandrine Le Bail^3^, Jean-Louis Couderc^3^ and Muriel Grammont^1,2^

In the figure 2 of the publication, the cells 1, 2, 3, 4, 5 and 63 correspond to the cells numbered 3, 9, 19, 27, 25 and 63, respectively, by the macro when running on the test stack.

**Macro Image J (iMetrics); a test stack (test.tif) is provided.**

//Determination of the morphometrics of Drosophila follicular cells

//Auteurs : Anne Beghin, Centre Commun de Quantimetrie ; Muriel Grammont, UMR5534 Lyon

//Version 3.1, date 22/02/14

//Required : MBF_imageJ, 1.45 + plugins morphology Landini

macro "iMetrics Action Tool - Ccb9D2bCdbaDa6Cdb9D2dCba9D93Dc3Dd3De3Cba8Db5Ca98Da4C986D85Ca87D2aD39D48D49D94Da3Db3Db4Dc4C643D67Ccb9Da5C433D69Ccb9D29CcbaDb6Cba9Dd5Cca8D95C333D89C643D87C764D75CcbaD96Cdb9D1aC643D43Cca8D1cCfffD11D12D13D14D15D16D17D18D19D21D22D23D24D25D26D27D28D2eD31D32D36D37D3dD3eD41D42D47D4cD4dD4eD51D52D58D59D5aD5bD5cD5dD5eD61D62D63D6bD6cD6dD6eD71D72D73D74D76D7cD7dD7eD81D82D83D86D8dD8eD91D92D97D9dD9eDa1Da2Da7Da8DaeDb1Db2Db7Db8Db9DbaDbeDc1Dc2Dc7Dc8Dc9DcaDcbDccDcdDceDd1Dd2Dd6Dd7Dd8Dd9DdaDdbDdcDddDdeDe1De2De4De5De6De7De8De9DeaDebDecDedDeeCcb9D2cCdbaD1eCcbaD4aC543D79CdcaD4bCba9Dd4Cb98D84C643D44C643D56C333D10D20D30D40D50D60D6aD70D7aD7bD80D8aD8bD8cD90D98D99D9aD9bD9cDa0Da9DaaDabDacDadDb0DbbDbcDbdDc0Dd0De0Df0Df1Df2Df3Df4Df5Df6Df7Df8Df9DfaDfbDfcDfdDfeC643D66Ccb9D1dC543D88Cb97D38Cba9Dc5C754D46CdbaD3aC753D77CcbaDc6C543D78C964D57C643D64Ccb9D3bC754D65CdcaD3cC643D00D01D02D03D04D05D06D07D08D09D0aD0bD0cD0dD0eD0fD1fD2fD33D34D35D3fD45D4fD53D55D5fD6fD7fD8fD9fDafDbfDcfDdfDefDffC643D68Cca8D1bC643D54"{

setBatchMode(true);

getPixelSize(unit, pixelWidth, pixelHeight);

Start();

Orientation();

Analyse();

function ROInumerotate ();

function Start(){

run("Set Measurements...", " mean redirect=None decimal=3");

rename("originale");

setSlice(1);

run("Reduce Dimensionality...", " slices keep");

rename("Only_rouge");

selectWindow("originale");

setSlice(2);

run("Reduce Dimensionality...", " slices keep");

rename("Only_bleu");

}

function Orientation(){

//ParamËtres imageJ

run("Colors...", "foreground=black background=white selection=yellow");

//To get the shape of the follicle

selectWindow("Only_bleu");

nbr = nSlices;

run("Z Project...", "start=1 stop="+nbr+" projection=[Max Intensity]");

run("Unsharp Mask...", "radius=5000 mask=0.60");

run("Duplicate...", "title=MAX_Only_bleu-1");

setAutoThreshold("Li dark");

run("Convert to Mask");

run("Analyze Particles...", "size=2000-Infinity circularity=0.00-1.00 show=Masks exclude");

selectWindow("MAX_Only_bleu-1");

close();

//To calculate the angle of the follicle with the x axis

selectWindow("Mask of MAX_Only_bleu-1");

run("Options...", "iterations=8 count=4 edm=Overwrite do=Close");

run("Fill Holes");

run("Options...", "iterations=50 count=4 edm=Overwrite do=Close");

run("Set Measurements...", " fit redirect=None decimal=3");

run("Clear Results");

run("Analyze Particles...", "size=2000-Infinity circularity=0.00-1.00 show=Nothing display exclude add");

angle = getResult("Angle",0);

run("Clear Results");

print(angle);

//To Get extremities

roiManager("Select", 0);

drawFeretsTips(2);

run("Analyze Particles...", "size=0-Infinity circularity=0.00-1.00 show=Nothing add");

//To Get anterior extremity

run("Set Measurements...", " standard redirect=None decimal=3");

run("Clear Results");

selectWindow("MAX_Only_bleu");

roiManager("Select", 0);

run("Make Inverse");

run("Fill", "slice");

run("Select None");

intensity =0;

for(i=1;i<=2;i++){

roiManager("Select", i);

run("Enlarge...", "enlarge=100 pixel");

run("Measure");

currentintensity = getResult("StdDev",i-1);

if(currentintensity>intensity){

origin = i;}

intensity = currentintensity;

}

roiManager("Deselect");

run("Select None");

//To do the EDM

selectWindow("Tips");

run("Select None");

run("Duplicate...", "title=EDM");

roiManager("Select", origin);

run("Clear Outside");

run("Select None");

run("Invert");

EDM16b("Fire");

run("Calibrate...", "function=[Straight Line] unit="+unit+" text1=[1 2 ] text2=["+pixelWidth+" "+2*pixelWidth+"]");

//close

selectWindow("Tips");

close();

selectWindow("Results");

run("Close");

selectWindow("Mask of MAX_Only_bleu-1");

roiManager("Show None");

//Fonctions

function drawFeretsTips(pointsize) {

requires("1.29n");

diameter = 0.0;

getSelectionCoordinates(xCoordinates, yCoordinates);

n = xCoordinates.length;

for (i=0; i<n; i++) {

for (j=i; j<n; j++) {

dx = xCoordinates[i] - xCoordinates[j];

dy = yCoordinates[i] - yCoordinates[j];

d = sqrt(dx*dx + dy*dy);

if (d>diameter) {

diameter = d;

i1 = i;

i2 = j;

}

}

}

run("Colors...", "foreground=black background=white selection=yellow");

run("Select None");

run("Duplicate...", "title=Tips");

run("Grays");

run("Select All");

run("Clear", "slice");

run("Point Tool...", "mark="+pointsize+" selection=yellow");

makePoint(xCoordinates[i1], yCoordinates[i1]);

run("Draw");

makePoint(xCoordinates[i2], yCoordinates[i2]);

run("Draw");

run("Select None");

}

function EDM16b(lut){

run("Options...", "iterations=1 count=1 edm=16-bit do=Nothing");

run("Distance Map");

run("Set Scale...", "distance=1 known="+pixelWidth+" pixel=1 unit=µm");

run("Invert LUT");

run(""+lut);

selectWindow("EDM");

close();

selectWindow("EDM of EDM");

rename("EDM");

}

/*Eventually rotate follicle

if(origin == 1){angle = angle+180};

run("Select None");

selectWindow("Mask of MAX_Only_bleu-1");

run("Rotate... ", "angle="+angle+" grid=0 interpolation=None stack");

selectWindow("originale");

run("Rotate... ", "angle="+angle+" grid=0 interpolation=None stack");

selectWindow("Only_rouge");

run("Rotate... ", "angle="+angle+" grid=0 interpolation=None stack");

selectWindow("Only_vert");

run("Rotate... ", "angle="+angle+" grid=0 interpolation=None stack");

selectWindow("Only_bleu");

run("Rotate... ", "angle="+angle+" grid=0 interpolation=None stack");

selectWindow("MAX_Only_bleu");

run("Rotate... ", "angle="+angle+" grid=0 interpolation=None stack");

selectWindow("Tips");

run("Rotate... ", "angle="+angle+" grid=0 interpolation=None stack");

*/

}

function Analyse(){

//Getting the membranes

selectWindow("Only_rouge");

nbr = nSlices;

run("Z Project...", "start=1 stop="+nbr+" projection=[Max Intensity]");

run("Auto Local Threshold", "method=Median radius=10 parameter_1=0 parameter_2=0 white");

run("Grays");

//Mathematic morphometric tools

run("Invert");

run("Analyze Particles...", "size=500-Infinity pixel circularity=0.00-1.00 show=Masks");

selectWindow("MAX_Only_rouge");

close();

selectWindow("Mask of MAX_Only_rouge");

run("Options...", "iterations=1 count=7 pad edm=Overwrite do=Close");

runMacro("..//plugins//morphology//PruneAll.txt");

run("Options...", "iterations=15 count=5 pad edm=Overwrite do=Dilate");

run("Options...", "iterations=2 count=4 pad edm=Overwrite do=Erode");

run("Skeletonize");

runMacro("..//plugins//morphology//PruneAll.txt");

run("Invert");

run("Options...", "iterations=1 count=1 pad edm=Overwrite do=Erode");

roiManager("Reset");

run("Select None");

//Measures

selectWindow("Mask of MAX_Only_rouge");

run("Analyze Particles...", "size=50-1000 circularity=0.00-1.00 show=Nothing exclude add");

ROInumerotate();

selectWindow("EDM");

roiManager("Show All");

run("Set Measurements...", "area mean standard modal min centroid center perimeter shape feret's redirect=None decimal=3");

run("Clear Results");

roiManager("Deselect");

roiManager("Measure");

roiManager("Save", "..\\MBF_ImageJ\\RoiSet.zip");

setBatchMode(false);

updateResults();

//Put on Screen

roiManager("Open", "..\\MBF_ImageJ\\RoiSet.zip");

//selectWindow("Mask of MAX_Only_bleu-1");

//close();

selectWindow("originale");

setSlice(1);

run("Reduce Dimensionality...", " slices keep");

rename("Only_rouge");

selectWindow("Only_rouge");

run("Z Project...", "start=1 stop=4 projection=[Max Intensity]");

roiManager("Show None");

roiManager("Show All");

selectWindow("Results");

selectWindow("Only_rouge");

close();

run("Tile");

}

function ROInumerotate(){

for(i=0;i<roiManager("count");i++){

roiManager("Select", i);

roiManager("Rename", ""+i+1);

}

}
